# At the risk of repeating ourselves… Publishing data replication and negative data is good practice

**DOI:** 10.1002/phy2.273

**Published:** 2014-03-20

**Authors:** Mrinalini C. Rao

**Affiliations:** ^1^ Departments of Physiology & Biophysics and Medicine University of Illinois at Chicago Chicago Illinois

*Physiological Reports* is off to a great start. The wonderful outcome of collaboration between two major physiological societies, The Physiological Society and the American Physiological Society, its success is in no small measure due to the diligence of the members of our editorial board and the number of high‐quality submissions received. We are on our second volume and not quite a year old. We look forward to a robust 2014 with an increased presence in various social media outlets.

Our goal is to keep the discipline of physiology dynamic and bring to the reader the latest ideas in physiology “whose publication will be of benefit to the community” (Wray 2013). The Editor‐in‐Chief, Deputy Editor, and Associate Editors are continuously refining the parameters of “benefit to the community”—innovative science using cutting edge technology in unraveling fundamental questions, new ideas outside of conventional thinking, modeling based on solid evidence, well‐grounded findings that are contrary to the hypotheses, and confirmatory studies of previously published work with additional nuances. The latter two, also known as negative data and replication, are often frowned upon by mainstream peer‐reviewed journals as not being new, but we at *Physiological Reports* consider them a serious offering. This is a conundrum we all face as scientists—the first mantra we recite to our students is to make sure they can replicate their own findings sufficiently to stand statistical and scientific scrutiny. The second mantra is “let your data do the talking” and negative findings are important. A subset of negative findings is the inability to repeat what is accepted as dogma or established findings. Yet we as a profession balk at allowing our peers to make any of this available in a published format.

Validation of science is the ability to repeat the findings of others, even earlier avatars in one's own laboratory, and to build upon it. When findings of others cannot be replicated, especially repeatedly, the burden of proof nevertheless falls, not on the original authors, but on all the subsequent scientists. Current funding and, ergo, publishing pressures result in a number of us avoiding the issues, seeking alternate models or taking refuge behind explanations of compartmentalization, genetic differences in germ lines, dietary alterations, media differences, etc. While some of these may account for the observed differences, often times it is conceivable that the original paper had some loopholes that were not obvious at the time of publication. Consider the flip side—if a published finding cannot be repeated and there is no published knowledge about this, numerous hours and resources are wasted as different laboratories try to glean this information for themselves. In some cases this could have far reaching consequences, including altering the policies of regulatory agencies (see below). It is true that corrigenda are published if a gross error is made, but these are often buried and garner less attention. Therefore, we posit that *Physiological Reports*, with its easy access and turn around, should continue to be a venue to submit such replicative findings. As we were having this discussion, we recognize that *Nature Biotechnology* recently published an article with negative findings that refuted a published paper in *Cell Research* (Nature Biotechnology, 2013). The original finding reported the presence of plant microRNAs in human plasma and had far reaching consequences, including causing the Food Standards Australia New Zealand to publish a position statement on genetically modified crops. As outlined in the *Nature Biotechnology* editorial, there is an increasing interest by industry and funding organizations to provide opportunities, including commissioning studies, to evaluate reproducibility.

The importance of being able to replicate studies is also moving to the top of the agenda of funders such as NIHR and pharmaceutical companies. Several recent studies show the irreproducibility of many important experiments, again as noted in a *Nature* editorial (Wadman 2013), “In a 2011 internal survey, the pharmaceutical firm Bayer HealthCare of Leverkusen, Germany, was unable to validate the relevant preclinical research for almost two‐thirds of 67 in‐house projects. Then, in 2012, scientists at Amgen, a drug company based in Thousand Oaks, California, reported their failure to replicate 89% of the findings from 53 landmark cancer papers.” This has caught the attention of the popular press both in the *Economist* (2013) and in the *New York Times Sunday Review* (Chwe 2014); researchers are urged to confront our “biases.” As scientists and authors should we not welcome increased opportunities to contribute to the validation of important data and findings?

We at *Physiological Reports* would like to encourage our readership to include in their papers, where germane, their experiences with replicating the findings of others. Documenting in a sentence or two a positive replication and having a more nuanced discussion of negative findings would be of great use to the readership. We count on the integrity of our authors to submit their confirmatory studies with the context of why the information is important, and negative findings in a constructive and critical format that would engender healthy scientific dialog. That will certainly meet our goal of providing “a platform for all physiological research whose publication will be of benefit to the community.” We welcome your feedback.
